# The ANU WellBeing study: a protocol for a quasi-factorial randomised controlled trial of the effectiveness of an Internet support group and an automated Internet intervention for depression

**DOI:** 10.1186/1471-244X-10-20

**Published:** 2010-03-08

**Authors:** Kathleen M Griffiths, Dimity Crisp, Helen Christensen, Andrew J Mackinnon, Kylie Bennett

**Affiliations:** 1 Centre for Mental Health Research, School of Health & Psychological Sciences, College of Medicine, Biology and Environment, The Australian National University, Australia; 2 ORYGEN Research Centre, University of Melbourne, Australia

## Abstract

**Background:**

Recent projections suggest that by the year 2030 depression will be the primary cause of disease burden among developed countries. Delivery of accessible consumer-focused evidenced-based services may be an important element in reducing this burden. Many consumers report a preference for self-help modes of delivery. The Internet offers a promising modality for delivering such services and there is now evidence that automated professionally developed self-help psychological interventions can be effective. By contrast, despite their popularity, there is little evidence as to the effectiveness of Internet support groups which provide peer-to-peer mutual support.

**Methods/Design:**

Members of the community with elevated psychological distress were randomised to receive one of the following: (1) Internet Support Group (ISG) intervention, (2) a multi-module automated psychoeducational and skills Internet Training Program (ITP), (3) a combination of the ISG and ITP, or (4) an Internet Attention Control website (IAC) comprising health and wellbeing information and question and answer modules. Each intervention was 12 weeks long. Assessments were conducted at baseline, post-intervention, 6 and 12 months to examine depressive symptoms, social support, self-esteem, quality of life, depression literacy, stigma and help-seeking for depression. Participants were recruited through a screening postal survey sent to 70,000 Australians aged 18 to 65 years randomly selected from four rural and four metropolitan regions in Australia.

**Discussion:**

To our knowledge this study is the first randomised controlled trial of the effectiveness of a depression ISG.

**Trial registration:**

Current Controlled Trials ISRCTN65657330.

## Background

It has been projected that depression will be the leading cause of disease burden in 2030 [1]. Lifetime prevalence of depression is high [2] and depressive episodes are frequently persistent and recurrent [3]. Moreover, only a minority of people with depression receive evidence-based treatment [4].

It is likely that the undertreatment of depression is due in part to lack of availability and accessibility of services [5], an unwillingness to seek help due to the stigma associated with depression [6] and a preference among some people with depression for employing self help methods for coping with the condition. One potential means for increasing treatment coverage and reducing burden is to facilitate the large scale dissemination of self help methods which reduce the need for the health workforce involvement and circumvent the stigma associated with seeking face-to-face help.

There is some evidence that self help interventions delivered online can be effective in reducing depressive symptoms [7]. In particular, a number of studies have reported a reduction in depressive symptoms associated with the use of automated online programs in the absence of clinical input (e.g., [8-14]). Online programs have also been demonstrated to reduce the stigma associated with depression [15,16] and to increase mental health literacy among people with elevated depressive symptoms [8,17]. Less is known about the effect of these programs on other factors such as empowerment, quality of life, self esteem, disability, and service use. Further, although a frequently cited advantage of online psychological applications is their potential for use with under-served rural populations [18] there have been no direct comparative studies of the acceptability and effectiveness of these programs in rural compared to metropolitan regions [7].

Research into the effectiveness of online interventions for depression has been focused primarily on applications designed to deliver psychological skills training. However, the widespread availability of the Internet has stimulated the development of a large number of online 'mutual support' or 'self help support' groups. Such support groups have been estimated by Eysenbach to be used by 'millions' of consumers daily [18] and there is evidence that they are particularly popular among consumers with depression [19]. However, the efficacy of such Internet support groups (ISGs) has not been established.

A recent systematic review of depression ISGs identified no randomised controlled trials of the effectiveness of the groups, for individuals with depression, in reducing depressive symptoms or in improving other psychological, mental health, service-related or other outcomes [20]. However, there was some evidence that peer-to-peer support groups might be associated with a reduction in depressive symptoms. In particular, in a prospective cohort study, Houston [21] reported a reduction over 6 months in depressive symptoms among high frequency users of public internet support groups after controlling for initial severity of symptoms. In addition, a systematic review of efficacy trials of ISGs for consumers with a range of health conditions yielded some encouraging evidence that such ISGs may be effective in reducing depressive symptoms among women with breast cancer [22].

It is possible however, that the primary effect of depression support groups is on outcomes other than symptomatology. For example, it is often claimed that support groups increase consumer empowerment and social support [23,24]. However, to date there have been no quantitative studies of the effect of depression ISGs on outcomes such as empowerment, self esteem, social support, quality of life or other factors such as depression knowledge and help seeking.

One potential limitation of fully automated online programs is the likelihood of substantial dropout during their unguided use [25]. Automated messages and reminders might reduce attrition rates in such circumstances [26]. It has also possible that participation in an ISG might facilitate adherence to online applications. Indeed, many online chronic disease management programs employ ISGs (e.g., see [20]). However, to our knowledge, to date there have been no systematic studies of the effect of online peer-to-peer support groups on adherence or outcomes among consumers receiving internet applications.

A final limitation of available research is that the majority of randomised controlled studies of e-mental health programs have employed wait list control groups [27]. A small number of studies have employed an attention control group or a psychoeducational controls. However, these studies have some limitations. For example, Christensen and her collaborators used a telephone attention control but the control group did not receive an internet intervention [8,9,15]. Some studies have used an online psychoeducational website as a control (e.g., [28-30]). However, a recent meta-analysis demonstrated that the provision of mental health information may reduce depressive symptoms among adults with depression or depressive symptoms [31]. To date no study of the efficacy of e-mental health intervention has employed an attention control condition which both comprised a website and contained plausible content not containing depressive or anxiety psychoeducational information.

### Objectives of the ANU WellBeing study

The primary objectives of the study are to (i) evaluate the efficacy of a depression Internet Support Group (ISG) and an automated psychoeducational and skills Internet Training Program (ITP) for reducing depressive symptoms relative to a plausible Internet Attention Control condition (IAC); to evaluate the relative efficacy of the ITP, ISG and combined ITP and ISG interventions; and (iii) to ascertain if the adherence rates and magnitude of improvement in depressive symptoms is greater for the ISG and ITP interventions combined than for either condition alone.

Secondary objectives of the study are to (i) evaluate the effect of the two experimental interventions on anxiety, disability, social support, loneliness, self-esteem, empowerment, loneliness, depression literacy, stigma and help seeking in people with elevated depressive sympotms; and (ii) to compare the outcomes of these interventions in rural and metropolitan residents.

## Methods/Design

### Trial Design

Members of the community with elevated levels of depressive symptoms were randomised in a 2 by 2 quasi-factorial design to receive either the ISG, the ITP, a combination of the ISG and IPT or a plausible internet-based attention control (IAC) (see Table 1). Measures were administered at screening, baseline, post-test, and 6 and 12 months after the intervention commencement.

**Table 1:** Quasi-factorial trial design and target sample sizes per cell.

	Internet support group (ISG)		
**Automated skills training program (ITP)**	**No ISG**	**ISG**	**Target total**

**No ITP**	125 (IAC)	125	250

**ITP**	125	125	250

Target total	250	250	500

Approval to conduct the trial was granted by The Australian National University Human Research Ethics Committee (Protocol 2007/2259). The study protocol is registered with the Controlled Clinical Trials registry (ISRCTN65657330).

### Participant recruitment

Participants in the ANU WellBeing Study have been recruited via postal invitation and survey of 70,000 adults aged between 18 to 65 years who were randomly selected from the electoral rolls of four rural and four metropolitan Australian electoral divisions. Registration on the electoral roll is compulsory for all Australian citizens. Participants were selected from the division list using the 'select cases' random selection facility in SPSS. The metropolitan samples comprised registrants from one electoral division in Canberra, The Australian Capital Territory; two electoral divisions in Melbourne, Victoria (Maribyrnong, Higgins); and one division in Sydney, NSW (Bennelong). The rural divisions were located in Victoria (Indi, Murray) and New South Wales (Hume, Riverina). Divisions were selected from those which had a moderate (51%) to high proportion (>62.2%) of households with internet access [32]. Participants were oversampled in the rural divisions to adjust for lower internet usage in these regions.

The screening survey was accompanied by a letter and brochure which described both an enclosed 'WellBeing' screening survey and an intervention project ("The Wellbeing Promotion Study"). The participant was informed that the WellBeing survey was designed to 'find out more about the mental health of Australians in the city as well as in rural areas' and that the WellBeing Promotion Study was designed to 'look at the usefulness of self help internet programs for improving emotional well being and preventing or reducing the symptoms of depression'.

Survey respondents who met the eligibility criteria for the WellBeing study (see Table 2), including elevated levels of psychological distress as measured by the Kessler Psychological Distress (K10) scale, and a willingness to be contacted about participating in the trial were notified by letter that they would telephoned by a project interviewer to discuss the project. The assigned interviewer subsequently contacted the eligible respondent to provide further details about the trial including a brief description of the four conditions to which they might be randomly allocated, to provide information about the participant time commitment involved, to answer questions about the study, and to confirm that the respondent satisfied the inclusion criteria for the study.

**Table 2:** Inclusion and exclusion criteria for the WellBeing trial

Inclusion	Exclusion
Age 18 to 65 years	Self-reported current or imminent treatment from a mental health professional or mutual support group at the time of recruitment.

Kessler Psychological distress (K10) score > 22	Current treatment with Cognitive Behaviour Therapy.

Home or work access to the Internet	Self-reported current or past experience with or diagnosis of psychosis, schizophrenia or bipolar disorder.

Consent to participate	Current or prior participation in another project conducted by the lead investigator's research centre.

Eligible individuals who agreed on the telephone to participate in the project were randomised (see below) to one of the four trial conditions and were sent an information sheet about the project and the intervention conditions together with a consent form. At this point, however, neither they, their interviewer nor the project coordinator were informed of their randomisation status. Randomisation prior to consent and completion of the pre-intervention was undertaken for pragmatic reasons as the trial required the participants in the internet support groups to commence simultaneously. Undertaking stratified randomisation post-consent and post-baseline survey would have either introduced a significant delay between screening and baseline measures and the commencement of the intervention or reduced the available pool of participants.

Those participants who returned consent forms were sent a letter informing them of the condition to which they had been randomised together with a 14 or 15 page user guide containing a week by week overview of their intervention, a 'Frequently Asked Questions' section and a 'Technical Tips' page.

Recruitment to the WellBeing Study was conducted in three waves (Pilot, Wave 1, Wave 2) between August 2008 and May 2009. A subsample of 7000 surveys (randomly selected from the two metropolitan divisions of Canberra and Maribyrnong and the two rural divisions of Murray and Indi) were employed to pilot the survey and protocol. After the successful completion of the intervention phase of the pilot, a further 28,000 surveys were sent to residents in the above divisions. Since these screening surveys did not yield the target sample size for the study, a further 35,000 surveys were sent to the divisions of Bennelong, Higgins, Murray and Riverina.

### The Interventions

The study involved four intervention conditions comprising the two intervention programs presented singly and in combination and an attention-control website administered over a 12 week period. Although a module could not be accessed until the week scheduled for its delivery, it remained open for the remainder of the 12 week intervention period and for 12 months from the commencement of the intervention. Participants in the attention control condition were provided with access to the ITP after the 6-month follow-up assessment. The ITP, ISG and IAC conditions employed the e-couch, WellBeing Board and HealthWatch websites respectively. The weekly content of each intervention websites is summarised in Table 3 and described in detail below.

**Table 3:** Summary of the WellBeing Trial topics for the intervention and control conditions.

	Condition		
	
Week	ITP: e-couch	ISG: WellBeing Board	IAC (Control): HealthWatch
1	Depression: What is depression? Risks & causes	How do you feel? (*Your WellBeing category*)	Environment *(Questions)* Environmental Health *(Information)*
2	Treatments: What works for depression	What helps? Who helps? (*Feeling Better category*)	Nutrition *(Questions)* Nutrition Myths *(Information)*
3	CBT: Thinking about thinking. (identifying thinking patterns that can lead to depression)	General chit-chat (*General category*)	Physical & Mental Activities *(Questions)* Maintaining a Healthy Heart *(Information)*
4	CBT: Changing your thinking (changing your thoughts to change your mood)	Do they understand? (*Your WellBeing forum*)	Physical Health *(Questions)* Energize Yourself (& Your Family) *(Information)*
5	CBT: Changing your behaviour (how to change your mood by doing things differently)	Positive things that happened to you today. (*Feeling Better forum*)	Social & Family Relationships *(Questions)* Medicines in the Home *(Information)*
6	CBT review (work on your workbook)	Jokes (*General forum*)	Travel *(Questions)* Temperature Extremes *(Information)*
7	IPT: Role disputes and relationships	Causes and triggers (*Your WellBeing forum*)	Education & Post Education *(Questions)* Oral Health *(Information)*
8	IPT: Role changes and grief	Your views on antidepressants & Connecting with others (*Feeling Better forum*)	Career & Work Relationships *(Questions)* Blood Pressure & Cholesterol *(Information)*
9	Physical activity (Part 1)	Creative corner (*General forum*)	Music & The Arts *(Questions)* Stroke *(Information)*
10	Physical activity (Part 2)	Effects of stress and feeling bad (*Your WellBeing forum*)	Popular Culture & Movies *(Qs)* Bacteria & Foodborne Illness *(Information)*
11	How to relax	Psychological therapy & improving your self-esteem (*Feeling Better forum*)	Humour *(Questions)* Calcium *(Information)*
12	Review	Alternative and lifestyle approaches (*Feeling Better forum*)	Sport *(Questions)* Back Pain *(Information)*

#### ITP: E-couch (depression stream)

This automated online psychological intervention is a research version of the depression stream of the online application e-couch http://ecouch.anu.edu.au. The intervention comprised a depression literacy module together with five interactive self help tools designed to reduce depressive symptoms.

**Figure 1:**
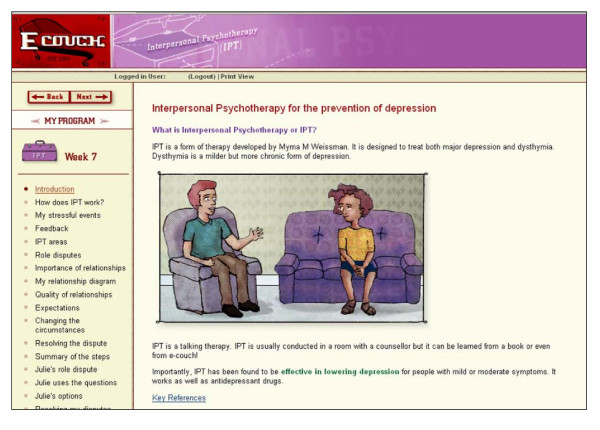
**Screenshot from the e-couch program week 7**.

The depression literacy module contained information about the symptoms and types of depression and their diagnosis, sources of help, information about the prevalence and disability burden associated with depression, depression risk factors, and evidence-based medical, psychological and lifestyle treatments for depression based on updated systematic reviews and clinical practice guidelines. The self help module comprised online versions of treatments amendable to delivery online and known to be effective in face-to-face therapy, bibliotherapy or computerised or Internet delivery. These included: (a) cognitive behaviour therapy; (b) interpersonal therapy; (c) applied relaxation; and (d) physical activity. An example page from the research version of e-couch is illustrated in Figure 1.

#### ISG: WellBeing Board

The WellBeing Board is a research ISG which uses a bulletin board format to facilitate discussions. It was developed for the trial based on the primary author's experience in running a public ISG and a thematic analysis of a randomly selected subset of topics on that board. The WellBeing board was divided into three main categories: Your Wellbeing, Feeling Better and General. Each week one, or in the case of weeks 8 and 11, two new forums or topics were introduced for each category in turn (see Table 3).

Participants were instructed to login to the website at least twice a week, read any new messages that had been posted, and contribute a minimum of four posts/messages a week. They were also encouraged to login to the WellBeing Board website as often as they wished, to introduce additional topics for discussion and to continue discussions across weeks.

A detailed list of the WellBeing board rules appeared when the participant first registered on the board and was available at all times on the Board home page and in the participant's manual. Discussion of suicide, self harm and other traumatic topics was not permitted. Participants were requested to use pseudonyms and to refrain from posting any material that might identify them. Links and email addresses were filtered out of posts and signatures using an automated script.

**Figure 2:**
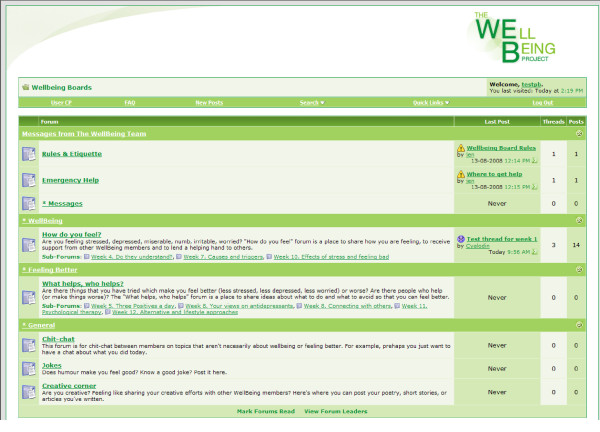
**Screenshot of the entry page for the WellBeing Board**.

The Board was moderated by a member of the WellBeing trial staff who enforced the rules but did not participate in the board discussion. The entry page of the WellBeing Board is illustrated in Figure 2.

#### ISG + ITP: WellBeing Board plus e-couch

The combined intervention involved the delivery of both e-couch and a dedicated WellBeing Board each week over the 12 week intervention period. The trial protocol including reminders was identical to that for the individual conditions. To prevent contamination across conditions separate ISGs were used for the combined intervention and ISG-only conditions. The combined condition ISG also differed from the single ISG intervention condition in that it incorporated an additional forum designed for participants to discuss their experiences on e-couch.

#### IAC: HealthWatch

In the absence of a suitable existing web-based attention control condition, the HealthWatch website was purpose built for the WellBeing trial. It comprised 12 modules, each consisting of two online components.

The first component involved a series of online questions probing factors potentially related to depression and wellbeing. This component was modified and extended to 12 modules from a previously reported 5 module telephone control condition [9,15,33]. For example, the introduction to the humour topic (Week 11) commenced with the explanation: "Some people believe that humour and laughter can prevent depression and increase the enjoyment of life. Today we would like to ask you to think about humour and whether it is important to your mood". This introduction was followed by a series of 16 multiple choice and open-ended questions: e.g., Do you enjoy telling jokes? (Responses: Yes, No, I can never remember any); How often do you tell jokes (Responses: Never, Rarely, About once a month, at least once a week, Most days); What is the funniest joke you can remember? (Open ended response); Overall do you think your moods are affected by humour?

The second component of the each weekly website module contained online information about a series of 12 topics related to wellbeing. The content in this component of the modules was adapted for Australian use from publications of the National Institute of Health and other US government sources which permit public use. Material was chosen which addressed topics relevant to wellbeing but which contained no or minimal information about interventions for depression or stress.

**Figure 3:**
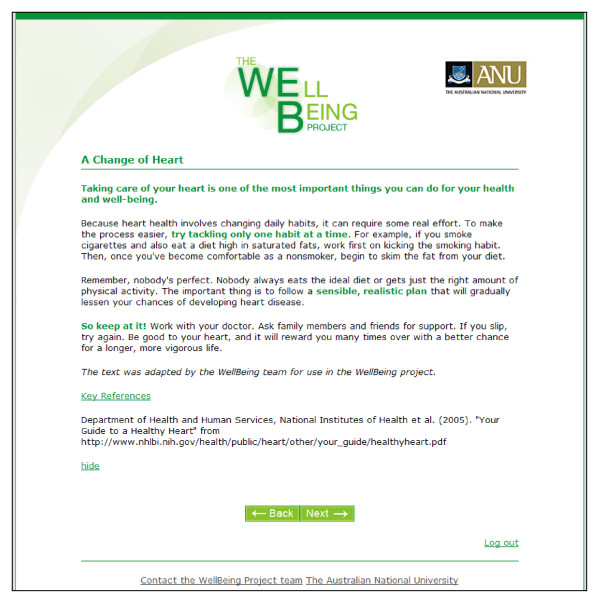
**Screenshot of a page from week 3 of the HealthWatch website**.

Participants were instructed to log onto the website each week to respond to the questions in first component of the module, and then to read the material in the second. An example page from HealthWatch website is shown in Figure 3.

### Intervention Study Procedure

All participants who provided written consent were sent a user ID and password by automated email. A second automated email sent one week prior to the commencement of the intervention directed them to log onto the website to complete the online baseline survey.

Each of the four conditions was then delivered over a period of 12 weeks. Participants received a weekly automated email informing them when the next module of their program was available. Those who failed to log onto the module were sent an email reminder four days after the initial email. Those who failed to log in thereafter received a telephone reminder from their interviewer. The current week's module was only available after first completing the modules assigned in the preceding week or weeks.

**Figure 4:**
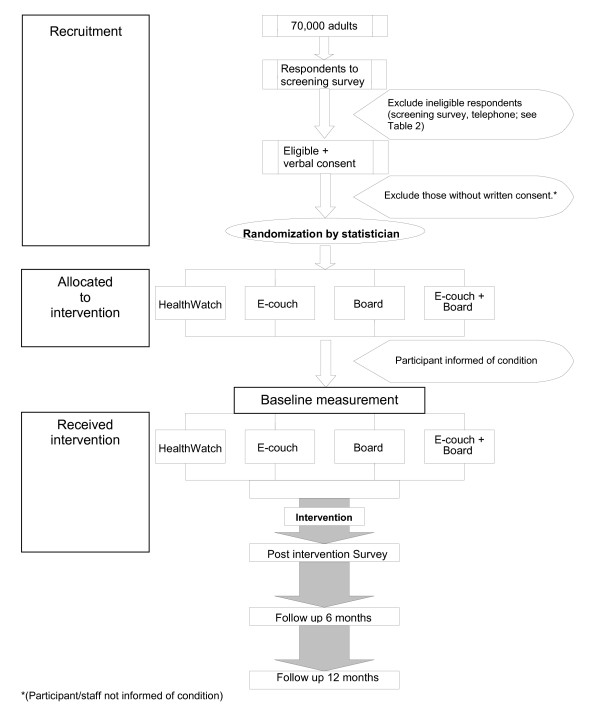
**Participant flow through the study**.

At the end of the 12-week trial, an automatic email was sent to participants requesting that they complete the post-intervention survey. Reminders were sent according to the protocol employed at baseline. Further follow up using this protocol was undertaken at 6 months and 12 months after the commencement of the intervention. The flow of participants is shown in Figure 4.

Participants could withdraw from the study without explanation at any stage by informing the Trial Manager or their interviewer via email or telephone.

### Randomization and allocation concealment

#### Allocation concealment

As required by ICH guideline E9 [34], randomisation was carried out by the trial biostatistician who was not involved in the day-to-day conduct of the trial.

#### Randomisation sequence

Potential participants were randomised using a stratified block design with stratification by level of psychological distress (high/low distress level on K10), gender (male, female, uncommitted), and location of residence (metropolitan/rural). Randomization schemes with a fixed block size of four were generated for each strata using the web site Randomization.com http://www.randomization.com.

### Measures

Table 4 provides a summary of measures taken at each assessment point.

**Table 4:** Measures employed in the screening and baseline, post intervention and follow-up phases of the WellBeing Study

	Screening	Baseline	Post-test	6 month	12 month
Depressive symptoms (CES-D)		X	X	X	X

Anxiety symptoms (PSWQ)		X	X	X	X

Quality of Life		X	X	X	X

Disability		X	X	X	X

Self-esteem		X	X	X	X

Perceived social support		X	X	X	X

Loneliness		X	X	X	X

Depression knowledge		X	X	X	X

Stigma	X	X	X	X	X

Help-seeking behaviours		X	X	X	X

Empowerment		X	X	X	X

User perceived utility of the Internet		X	X	X	X

Satisfaction & user perceived benefits/negative effects of intervention			X	X	X

Participation in healthcare			X		

Sociodemographics	X				

Psychological distress (K10)	X				

User perceived mental health status	X				

Depression & general anxiety symptoms (DASS-21)	X				

Social anxiety screening (BSPS)	X				

Panic screening	X				

Information needs	X				

Intervention group preference	X				

#### Primary outcome measure

*Depressive symptoms: *The primary outcome measure was the Centre for Epidemiological Studies Depression scale (CES-D; [35]). This 20-item self-report scale was used to measure change in the severity of depressive symptoms.

#### Secondary outcome measure(s)

The following secondary measures were used at *baseline *and in at least one *subsequent *survey to evaluate improvement or change in mental health, other outcomes and potential moderating or mediating factors:

*Anxiety severity*: The Penn State Worry Questionnaire (PSWQ)[36]; *Quality of life*: The EUROHIS QOL - 8 item index [37]; *Disability: *'Days Out of Role' questions adapted from the US National Comorbidity Survey [38]; *Self esteem: *Rosenberg Self-esteem Scale [39]; *Perceived social support: *The 8-item Medical Outcomes Study Social Support Survey (MOS)/Emotional/informational support subscale [40]. This scale was considered more relevant than others to online as opposed to face-to-face or telephone support. *Loneliness*: The UCLA Loneliness Scale [41]; *Stigma*: Depression Stigma Scale (DSS-Personal), a measure of personal, negative attitudes to depression [15,42]; *Depression knowledge*: (i) a short 11-item version of the Depression Literacy Scale (D-Lit depression literacy scale; [15]); (ii) 33 depression treatment knowledge items evaluating the perceived helpfulness ('Helpful', 'Neither helpful nor harmful', 'Harmful', or 'Don't know') of three medications, five psychological treatments 21 lifestyle or alternative treatments and 4 formal and informal sources of help [8,43]; *Help seeking: *Participants indicated (Yes or No) which of the above medication, psychological, lifestyle and alternative treatments or activities they had used or people they had consulted for depression in the past 3 months [8,43]; *Empowerment: *Power-powerlessness subscale of the Empowerment Scale [44]. This scale was developed in conjunction with mental health consumers; *Perceived utility of the Internet*: Two questions assessed participant's beliefs about the utility of the Internet for preventing and understanding depression ("I am confident that people could learn skills for preventing depression from a website" (Yes, No); "I am confident that a website could help people understand depression better" (Yes, No)).

In addition, the following measures were employed at one or more points post-intervention with a view to assessing their experience of the program: *Satisfaction and perceived benefits/negatives: *Satisfaction with the interventions was measured using 10 previously employed self-report items (e.g., 'How useful was the website?' (Very useful, useful, Not very useful, Not at all useful)) together with a series of questions based on items developed in a UK study of depression ISG users [45] (e.g., 'The website helped me to discuss subjects that I felt unable to discuss elsewhere.' (Strongly agree, Agree, Neither agree not disagree, Disagree, Strongly disagree) and the Consumer Reports CRES-4 scale [46,47]; *Perceived negative effects *were measured with items specifically developed for the purposes of this study (e.g., 'Were any of the following a problem for you as a result of using the website? 'Feeling annoyed or upset by the comments made by other members on the board.' (Yes, No).); *Participation in healthcare*: A three item measure, modified from a study of a physical health internet support group [48] was employed at post intervention to assess participants' self-reported capacity to discuss treatments with their provider, feel in control and knowledgeable and be assertive with their healthcare provider; *Adherence*: measured in two ways for each individual: (i) completion of post-test surveys; (ii) number of modules completed (e-couch & Health Watch); (iii) frequency of logins and number of weeks logged onto the WellBeing ISG and activity level on each website (*e-couch*: number and percentage of exercises completed; number and percentage of pages downloaded; *WellBeing Board*: number of posts, number of threads started; HealthWatch: number and percentage of questions completed and pages downloaded).

#### Demographic and other measures

A number of other measures were collected in the screening survey. These included *demographic *data including gender, age, postcode, marital status, size of town of residence, level of primary and secondary education completed, current study being undertaken, and employment (status, main activity, usual job, past job). In addition, *severity of general anxiety, depression and stress *was measured using The Depression Anxiety Stress Scale (DASS-21; [49]), *social anxiety symptoms *were measured using the fear and avoidance subscales of the Brief Social Phobia Scale (BSPS; [50]) and *panic symptoms *were measured using The 5-item Patient Health Questionnaire (PHQ - Panic; [51]) scale. Participant *perceived mental health status *(stress, anxiety, panic, social anxiety, or depression), *self reported professional help seeking *for any such problem and *perceived usefulness of seeking professional help *for these problems were also collected. A series of purpose designed items were included in the screening survey to assess participant self reported *need for information about depression*. Finally, an item was included to measure participant *randomisation preference *at baseline.

### Planned data analysis and sample size

The WellBeing trial aimed to recruit and randomise 500 participants to four conditions (125 per group). The primary analysis will be undertaken on an intent-to-treat basis. Mixed-model repeated measures (MMRM) analyses will be used because this method has been demonstrated to be superior to using the last observation carried forward method for handling missing data in longitudinal designs [52]. For analyses of categorical outcomes, non-linear mixed modelling methods will be used. Based on previous studies, [8] we estimated pre-post effect sizes for the automated training program, the attention placebo and the ISG to be .6, .1, and .35 respectively. Power was set at 80% and calculations conservatively assumed a correlation of .5 between pre- and post-test measurements. Under these conditions the target sample size could detect a change from baseline of approximately .25 standard deviations in *a priori *contrasts of treatment arms conducted within the framework of omnibus test of condition by time mixed model repeated measures analysis. It is planned that the unique effect of the combined treatments will be tested by appropriate contrasts. It is possible that the combinations of treatment (e-couch and ISG) will prove no better than the individual programs. Conversely, cumulative effects or potentiation (synergy) may be observed. The target sample size ensured that the study is powered to detect an effect of .25 in either direction.

## Discussion

To our knowledge this study is the first RCT of the effectiveness of a depression ISG, the first to systematically investigate if participating in a peer-to-peer ISG improves adherence and outcomes and the first to investigate the comparative effectiveness for rural and metropolitan consumers of an automated psychological intervention. In addition, there have been no previous studies of effect of an automated online self-help program on consumer empowerment.

A second novel contribution of this trial is its use of a purpose constructed plausible, web-based attention control containing authoritative information. At the time of writing, we were not aware of any published RCTs of the efficacy of e-mental health interventions which employed such a control. As already noted, depression or mental health education websites are not the preferred control condition for studies of interventions for mental disorders as there is evidence that such information may improve mental health. We did consider referring control group users to existing websites with a 'wellbeing' theme. However, these websites were typically characterised by one or a number of limitations in that they contained content that: included potentially active components (such as exercise interventions); incorporated advertisements, which raises ethical issues when used in the context of a research trial; were typically not evidence-based, a further ethical concern; might change or be removed from the Internet; and was not subject to the control of the research team. Moreover, external websites could not be seamlessly integrated into our automated research trial processes and accordingly it would not have been possible to automatically track trial participant access and usage of these sites.

### Status of the Trial

The trial was commenced in August 2008. Participants recruited in the pilot and wave 1 stages of the study have completed the 12-month follow-up. Participants recruited in the wave 2 stage have completed the 6-month follow-up with 12 month follow up due to be completed in May 2010.

## Competing interests

KG and HC are directors of e-hub at the ANU which developed the E-couch program and runs depression and anxiety internet support groups. However, neither author derives personal financial benefit from the operation of e-hub.

## Authors' contributions

KG conceived, designed and supervised the study, and drafted the paper. DC drafted the paper and served as trial manager of the study. HC designed the study and edited the paper. AM designed the study, provided statistical and methodological advice, undertook the randomisation process and edited the paper. KB contributed to the design of the protocol, oversaw the IT implementation of the project and edited the paper. All authors have read and approved the final manuscript.

## Pre-publication history

The pre-publication history for this paper can be accessed here:

http://www.biomedcentral.com/1471-244X/10/20/prepub
